# Accurate Diagnosis of *Pseudomonas aeruginosa* Is Critical to Mitigating Development of Antibiotic Resistance

**DOI:** 10.3390/antibiotics14050509

**Published:** 2025-05-15

**Authors:** Hala I. Al-Daghistani, Lubna F. Abu-Niaaj, Sima Zein

**Affiliations:** 1 Department of Medical Laboratory Sciences, Faculty of Allied Medical Sciences, Al-Ahliyya Amman University, Amman 19328, Jordan; h.aldaghistani@ammanu.edu.jo; 2 Department of Agricultural and Life Sciences, College of Engineering, Science, Technology and Agriculture, Central State University, Wilberforce, OH 45384, USA; 3 Department of Pharmaceutical Biotechnology, Faculty of Allied Medical Sciences, Al-Ahliyya Amman University, Amman 19328, Jordan; s.zein@ammanu.edu.jo

**Keywords:** *Pseudomonas aeruginosa*, antibiotic resistance, virulence factors, pyocyanin

## Abstract

**Background**: The accurate and rapid diagnosis of infections is critical for effective and timely treatment. Misdiagnosis often leads to the prescription of antibiotics not targeting the causing agent of infection and thus be the possible development of multidrug resistance. This collectively worsens the condition and might lead to unnecessary intervention or death. The abundance of *Pseudomonas* spp. in healthcare-settings and the environment may lead to the inaccurate diagnosis of *P. aeruginosa*, making the treatment of its infections challenging. *P. aeruginosa* is a Gram-negative, opportunistic pathogen commonly linked to healthcare-associated infections. Its pathogenicity is attributed to several virulence factors correlated to enhanced survivability and colonization, invasion of the host tissues, and the development of multidrug resistance. When advanced diagnostic facilities are limited or unaffordable, the prescription of antibiotics solely relies on identifying the bacteria by culture-based methods. **Objectives**: This study aims to validate the accuracy of diagnosis of fifty clinical isolates preidentified as *P. aeruginosa* in three healthcare facilities in Jordan. **Methods**: The isolates were from infected areas of patients, including skin, wounds, ears, urine, and peritoneal cavities. Morphological and biochemical tests were performed, and the validation relied on the polymerase chain reaction (PCR) amplification of the 16S ribosomal ribonucleic acid (rRNA) gene. This molecular method is affordable for medical facilities with limited finances in contrast to advanced high-cost techniques. **Results**: The PCR confirmed that only 60% of the isolates were *P. aeruginosa*. All the confirmed isolates could produce different pigments and form biofilms. **Conclusions**: The high percentage of isolates mistakenly identified as *P. aeruginosa* raises concern about the suitability of prescribed antibiotics. The present study strongly recommends using advanced molecular methods to identify the pathogens. If conventional methods remain the only diagnostic option, this study recommends frequent external validation for tests in addition to performing an antibiotic susceptibility test to pinpoint the effective antibiotics against biofilm-producing *P. aeruginosa.*

## 1. Introduction

*Pseudomonas aeruginosa* is a Gram-negative, rod-like, opportunistic pathogen causing a wide range of infections of the skin, lungs, and bloodstream. As typically found in healthcare settings, it poses a serious risk to vulnerable patients, especially those needing long-time care in hospitals for conditions including burns and wounds. It causes ventilator-associated pneumonia and post-surgery infections [1,2,3]. The bacterial adaptability to stressful environmental conditions is attributed to the expression of virulence factors, including exotoxin A, rhamnolipids (glycolipid biosurfactants), siderophores, pigments, in addition to several enzymes such as elastase, alkaline protease, and lipase [4,5]. The capability for biofilm formation and the development of antibiotic resistance are additional contributing factors to the increased pathogenicity and challenging treatment of infections [6,7].

A systematic analysis report by the Institute for Health Metrics and Evaluation (IHME) and the World Health Organization listed *P. aeruginosa* among the pathogens considered as a global burden due to their transmissibility, treatability, and prevention [8,9]. It was estimated that *P. aeruginosa* was responsible for 559,000 deaths annually worldwide, of which over 300,000 were associated with antimicrobial resistance [10]. Globally, an estimated 700,000 deaths annually are currently attributable to antimicrobial resistance (AMR), with a projected estimate of 10 million deaths by 2050 and an economic loss up to 100 trillion USD per year [11]. In the United States, the Centers for Disease Control and Prevention (CDC) estimated that *P. aeruginosa* caused 32,600 hospitalized infections with 2700 deaths in 2017, with an increase of 32% from 2019 to 2020. In 2024, the CDC listed *P. aeruginosa* among the top seven antimicrobial-resistant pathogens typically found in healthcare settings [12,13,14]. The Jordan National Antimicrobial Resistance report listed *P. aeruginosa* among eleven bacteria and fungi of public health and clinical importance [15]. A study focused on the Middle East and North African regions reported that the prevalence of multidrug-resistant *P. aeruginosa* identified in clinical samples was 52.5% [16].

The rise in AMR necessitates investigating the causes of this problem. The misuse of antibiotics is recognized among the reasons for the development of antibiotic resistance (AR) in bacteria. The key to avoiding AMR is accurate and rapid diagnosis to allow the prescription of the appropriate antibiotic. This is also critical to reducing recovery time and high medical costs [13,14,16]. On the other hand, it is critical to discover new antimicrobial agents from natural or synthetic resources. Several studies reported the growth inhibition of *P. aeruginosa* by several plants [17,18,19], while others documented the development of new synthetic compounds such as silver nanoparticles (AgNPs) to be used alone or in combination with standard antibiotics to inhibit the growth of *P. aeruginosa* [20,21].

The identification of *P. aeruginosa* is challenging using culture-based approaches due to the time needed for bacterial growth, with the potential for misidentification with closely related Gram-negative species. The polymerase chain reaction (PCR) technique has been widely used to diagnose and identify many pathogens (as was the case in an Iraqi journal), though the use of conventional culture methods is commonly applied in small-size clinical facilities due to affordability. Several new advanced techniques for microbial identification, including *P. aeruginosa*, have been developed. Among these techniques are Vitek/mass spectroscopy (MS), nuclear magnetic resonance spectroscopy, matrix-assisted laser desorption/ionization–time-of-flight MS, isothermal amplification, and next-generation sequencing [20,22,23]. The high cost and technical requirements of such techniques limit their application to research and reference laboratories [22]. Therefore, an accurate diagnosis of *P. aeruginosa* can be challenging when advanced diagnostic facilities are limited, especially when standardized national diagnostic protocols and monitoring policies are lacking [6,7]. Such limitations have led diagnostic facilities to solely rely on culture-based approaches and analytical profile index assays with a potential for misdiagnosis. This causes the prescription of improper antibiotics, which contributes to AMR development in *P. aseruginosa* [23].

The primary aim of this study is to use the PCR to validate the identification of clinical isolates prediagnosed in three healthcare facilities in Jordan as *P. aeruginosa.*

## 2. Results

### Detection of P. aeruginosa in Clinical Samples

**Figure 1:**
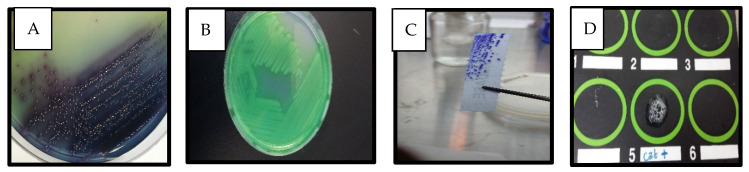
Characterization of *P. aeruginosa*. (**A**) Appearance on chromogenic agar, (**B**) pigment production, (**C**) oxidase test, (**D**) catalase test (positive in #5).

A total of 50 clinical samples received from three Jordanian healthcare facilities were initially identified as *P. aeruginosa.* This study validated that only 30 of the 50 isolates (60%) were *P. aeruginosa* based on the PCR amplification of the 16S rRNA gene. The cells of these isolates were Gram-negative rods, motile, and non-lactose fermenters. Colonies had a distinctive odor and grew on cetrimide agar, which is selective for *Pseudomonas.* All isolates were producers of oxidase, catalase, and nitrate reductase, though only 93.3% of them were gelatinase producers (Figure 1). It was observed that the isolates producing pyocyanin had a higher level of oxidase and catalase activity.

**Figure 2:**
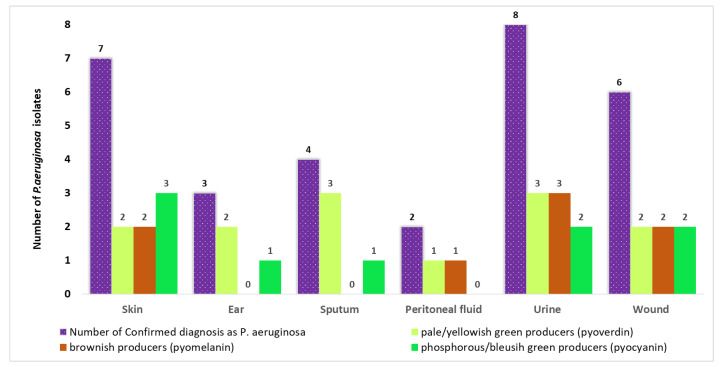
The distribution of isolates producing different pigments in comparison to the total number of isolates confirmed to be *P. aeruginosa* in the clinical samples received.

The validated *P. aeruginosa* isolates shown in Figure 2 were from urine (26.7%), skin (23.3%), wounds (20%), sputum (13.3%), ears (10%), and peritoneal fluid (6.7%). All of the isolates were pigment producers of different colors and shades.

**Figure 3:**
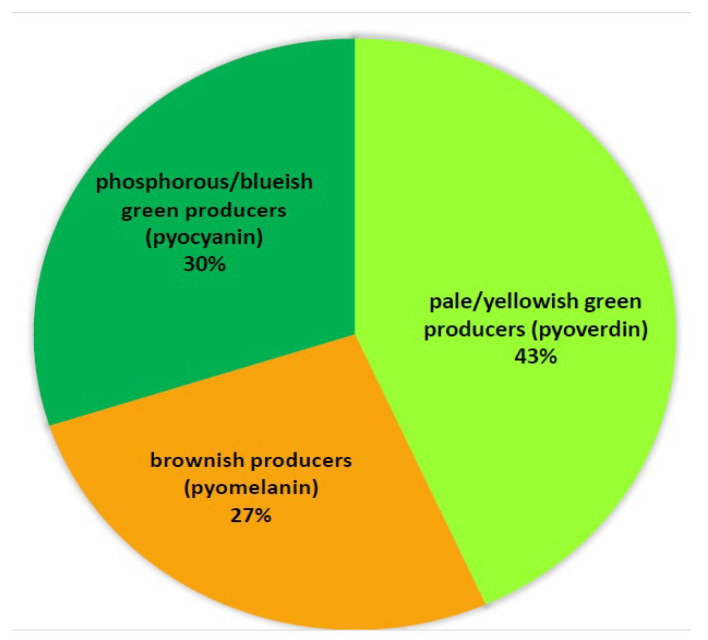
The categories of pigments produced by the confirmed *P. aeruginosa* isolates.

The colors of the produced pigments were categorized into three main groups: a yellow/pale yellowish green, which is a characteristic of pyoverdin; a brown pigment, which is a characteristic of pyomelanin; and a phosphorous/blueish green, which is a characteristic of pyocyanin (Figure 3).

Table 1 shows the number of isolates producing pyocyanin as follows: three out of seven skin isolates (42.8%), one out of three ear isolates (33.3%), two out of six wound isolates (33.3%), two out of eight urine isolates (25%), and one out of four sputum isolates (25%). None of the isolates of the peritoneum was capable of producing pyocyanin. The concentration of pyocyanin varies, showing the highest level of 36.448 μg/mL, which was produced by the sputum isolate, followed by an ear isolate (13.692 μg/mL), while the lowest concentration (0.171 μg/mL) was produced by one of the two wound isolates.

**Figure 4:**
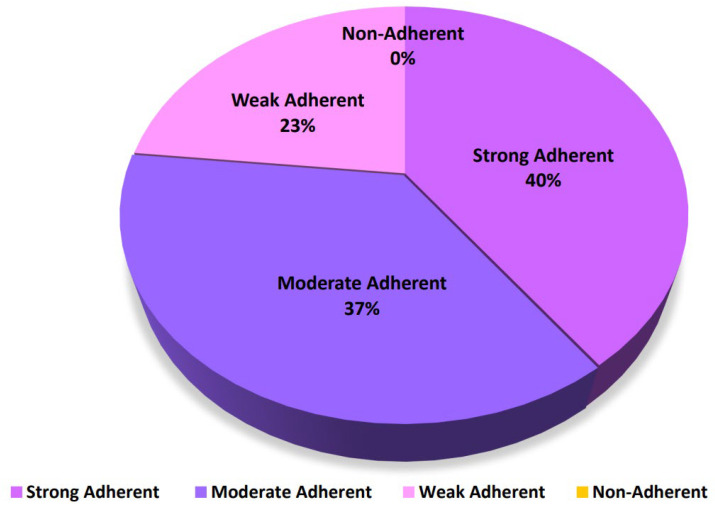
The adherence strength of the validated thirty isolates of *P. aeruginosa forming biofilms*.

A qualitative assessment of the capability of isolates to form biofilm was conducted for the 30 isolates confirmed as *P. aeruginosa*. According to the calculations and criteria described (Section 4.2), Figure 4 shows that 40% (12/30 isolates) of isolates were strong biofilm producers, 36.7% (11/30 isolates) were moderate biofilm producers, and 23.3% (7/30 isolates) were weak biofilm producers.

**Figure 5:**
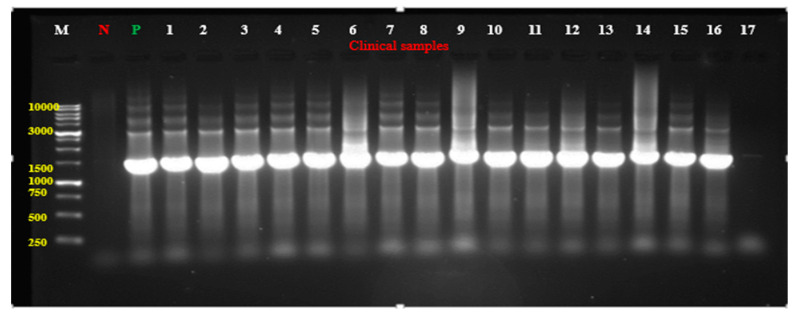
Agarose gel electrophoresis for the amplified *P. aeruginosa* DNA extracted from the clinical isolates (Lanes 1–17); M-Lane: 10Kb DNA ladder; N-Lane: negative control; P-Lane: positive control (*P*. *aeruginosa* ATCC 27853).

The PCR of the 16S rRNA gene was performed to validate the identity of the bacterial isolates as *P. aeruginosa.* In the amplification of the 16S rRNA gene, the gel electrophoresis for the PCR product revealed that ONLY 30 clinical isolates showed a band at 1451 bp which matches the size of the expected PCR product. The appearance of this band was indicative that the isolates were *P. aeruginosa.* Figure 5 shows the band as it appears for isolates (1–17). 

## 3. Discussion

The management of *P. aeruginosa* infections is facing significant obstacles due to the emergence of antibiotic-resistant strains. This phenomenon, along with other virulence factors, particularly biofilm formation, contributes to the bacterial pathogenicity by enhancing survivability, colonization, and invasion of the host tissues [24,25,26]. The proper, timely prescription of antibiotics depends on accurate and fast diagnosis of the infectious agent, which can be challenging when resources are limited as is the case in developing countries and rural areas. The present study aims to validate the diagnosis of 50 *P. aeruginosa* clinical isolates received from three clinical facilities in Jordan by relying on culture-based methods for microbial identification. The results of the phenotypic and biochemical tests are typical for *Pseudomonas* species. The validation of diagnosis relied on the PCR for the amplification of the 16S rRNA gene because it is a quick and affordable molecular method that targets desired genes. Our results showed that only 30 out of 50 isolates (60%) were diagnosed correctly as *P. aeruginosa.* The isolates were capable of producing colored pigments named pyoverdin, pyocyanin, and pyomelanin which are characterized by a pale/yellowish green, blueish/phosphorous green, and brown color, respectively. Studies showed that these pigments can enhance the pathogenicity of *P. aeruginosa* and contribute to antibiotic resistance, particularly in biofilm-producers [27]. Pyocyanin is a peculiar green pigment that has been studied exclusively to understand its role as a virulence factor in *P. aeruginosa.* The green pigment produced by the nine isolates was of different shades and the predominance of the yellow color is usually due to pyoverdin, which is also linked to bacterial resistance to various antibiotics.

Pyocyanin is a critical virulence factor that enhances bacterial pathogenicity, and it significantly exacerbates the severity and duration of bacterial infection [28]. A study focused on studying cystic fibrosis reported that isolated *P. aeruginosa* can produce pyocyanin in high quantities, suggesting its role in disrupting the movement of respiratory cilia, compromising host epithelial cell function, increasing mucus production, facilitating bacterial colonization, and intensifying inflammatory response [29]. Our study reports that the concentration of pyocyanin was in the range of 0.171 μg/mL to 36.448 μg/mL. The variation in its production among samples collected from the same body parts of different patients indicated no correlation to the source of samples. It is most likely that the high concentration is associated with the pathogenicity of *P. aeruginosa* causing the infection. A previous study reported that the highest concentration of pyocyanin was produced by a sputum isolate (>10 μg/mL) [26,28,29], though this concentration is not comparable to that secreted by the top two isolates reported for this study. Pyocyanin can act as both a redox-active secondary metabolite and a quorum-sensing signaling molecule inducing the production of reactive oxygen species, which causes DNA damage, disruption of diverse cellular processes, iron sequestration, and increased expression of virulence expression [28,29,30,31,32]. It maximizes the detrimental impact on host cells, as demonstrated in several studies highlighting a possible correlation between pyocyanin production and biofilm formation in *P. aeruginosa*. This relationship can be via the indirect promotion of the release of extracellular polymers such as polysaccharides, proteins, and lipids, fostering cellular aggregation and or it might be due to the dysregulation of quorum sensing [33,34,35]. The transition of *P. aeruginosa* to a mucoid phenotype during infection by the production of an exopolysaccharide allows the formation of biofilms by facilitating the production of a slime layer that enables strong adherence of cells to surfaces and to each other [36,37]. Biofilms are bacterial aggregates adaptive to environmental stresses and resistant to antimicrobial penetration [38]. The alginate layer of the biofilm-forming strain impedes the host’s optimal immune function by masking antibody opsonization and inhibiting the clearance of pathogens [39,40]. Several studies reported that the increased expression of genes encoding extracellular factors causes the development of acute infections.

Our study highlights the importance of validating culture-based diagnosis by molecular techniques to provide an accurate treatment intervention. As this might be a challenge to most healthcare facilities in developing countries, the validation can be achieved using the PCR for the amplification of specific 16S rRNA genes, which was reported to be specific in *P. aeruginosa* [41]. Using this basic molecular technique could be helpful to properly diagnosing infectious agents that are monitored due to global concerns over their development of resistance to standard antibiotics.

## 4. Materials and Methods

All chemicals were purchased from Thermo Fisher Scientific (Waltham, MA, USA), unless mentioned otherwise.

### 4.1. Sample Collection

A total of 50 clinical pure isolates grown on blind-labeled blood and MacConkey agar plates were received from three healthcare facilities in Jordan. The isolates were diagnosed at the facilities as *P. aeruginosa* based on culturing methods. The isolates were obtained from infected human skin, sputum, urine, wounds, ears, and peritoneal fluid. The study was approved under IRB: BAU/24/11/2022-2023.

#### 4.1.1. Biochemical Characterization

An overnight bacterial culture of each isolate was grown in the shaking incubator (JP Selecta, Barcelona, Spain) at 37 °C to be used for subculturing, morphological, and biochemical tests. The identification of the isolates as *P. aeruginosa* was validated by the amplification of the 16S rRNA gene. The subcultures on nutrient agar were used for phenotypic and biochemical tests. In addition, bacteria were subcultured on Cystine–Lactose–Electrolyte-Deficient (CLED) agar, cetrimide agar, and chromogenic agar. The CLED agar differentiates between the lactose and non-lactose fermenter bacteria, while cetrimide is selective for *P. aeruginosa*. The agar plates were incubated for 24 h at 37 °C to allow bacterial growth. The secretion of selective enzymes, including nitrate reductase, gelatinases, catalase, and oxidase, was conducted as described below using an overnight (18–24 h) bacterial broth [42,43,44]. 

Nitrate reduction test: An inoculum of bacterial culture was added to a tube containing nitrate broth, and the test tube was incubated at 37 °C for 48 h. A drop of sulfanilic acid and a drop of α-naphthylamine were added to the culture. Any color change from light amber or clear to red indicates a positive nitrate reduction test.

Gelatin hydrolysis test: A heavy inoculum of an overnight bacterial culture was stab-inoculated into tubes containing solidified gelatin and incubated at 37 °C for up to a week, with daily check for gelatin liquefaction, which occurs at 28 °C and above. The gelatin hydrolysis was confirmed by immersing the tube in an ice bath for 15 to 30 min. If the liquefaction persists after exposure to cold temperature (ice bath), that indicates the bacterial cells were capable of producing gelatinase.

Oxidase test: A bacterial colony from a fresh agar plate was rubbed onto an oxidase disc. A color change from white to purple within 10 s indicates an oxidase activity.

Catalase test: On a slide, a drop of fresh bacterial culture and a drop of hydrogen peroxide (3%) were added and mixed well. The formation of gas bubbles indicates a positive test.

#### 4.1.2. Extraction of Pyocyanin

The isolates were cultivated in *Pseudomonas* broth at 37 °C for 48 h. The pigment-rich broth cultures were centrifuged at 5000 rpm for 20 min, and the supernatant was filtered through 0.45 µm membrane nitrocellulose filters. A chloroform was added to the pigment filtrate in a 2:1 ratio while mixing for 30 s, and then the tube was allowed to stand for 10 min to separate the layers. The bottom blue layer was removed and extracted with 1 mL of 0.2 N HCL solution with gentle mixing until the color changed to pink. The last step of purification was repeated three times to ensure the high purity of pyocyanin. The concentration of pyocyanin was determined by measuring its absorbance at 520 nm by a spectrophotometer (Shimadzu-1700, Kyoto, Japan) and multiplying the absorbance value by 17.072 [45].

#### 4.1.3. Molecular Identification by Polymerase Chain Reaction (PCR)

The expression of the 16S rRNA gene was determined by the PCR. The extraction of the genomic DNA of *P. aeruginosa* was conducted according to the phenol/chloroform method. Briefly, a 1.5 mL fresh bacterial suspension in brain heart infusion broth was centrifuged, and the pellet was lysed in a mixture of 10% sodium dodecyl sulfate (SDS), Tris–EDTA (10xTE) buffer, sodium chloride (NaCl), and CTAB buffer (hexadecyltrimethylammonium bromide), mainly to remove the polysaccharides and polyphenols. Next, 10 µL of Proteinase K was added, and the suspension was incubated at 37 °C for 30 min. A mixture of phenol/chloroform/isoamyl alcohol (25:24:1) was added, and the solution was left to settle for 10 min before centrifugation at 16,000× *g* for 5 min. The DNA precipitate was obtained by adding a 0.6–0.7 volume of isopropanol then centrifuged for 15–30 min at 10,000–15,000× *g*. The supernatant was discarded, and the DNA pellet was suspended in 40 µL of RNase-free water and incubated at 37 °C for 1 h. The primers used to amplify the 16S rRNA gene were designed according to [46] (Humanizing Genomics Macrogen, Seoul, Republic of Korea) as shown below: 

The forward primer-27F was 5′-AGAGTTTGATCCTGGCTCAG-3′.

The reverse primer-1392R was 5′-GGTTACCTTGTTACGACTT-3′.

The 25 µL reaction mixture contained 1 µL of DNA template, 1 µL of each primer, 12 µL of the master mix (Promega, Madison, WI, USA), and 10 µL of distilled water. The amplification was performed in the thermal reader (BIO-Rad, Hercules, CA, USA) for 35 cycles. Each cycle underwent initial denaturation at 95 °C for 5 min, denaturation at 94 °C for 1 min, annealing at 54.2 °C for 1 min, and an extension at 72 °C for 90 sec except in the final cycle, which was for 10 min. The integrity of the PCR product was assessed by gel electrophoresis using 1% agarose. To 100 mL of melted agarose, 3–5 µL of ethidium bromide (10 mg/mL) was added to allow the DNA visualization by the UV transilluminator gel documentation system (Gel Doc 2000, Bio-Rad, USA). The PCR product for each bacterial isolate was prepared by adding 5 µL of loading dye to 1 µL of the amplified DNA. After the agarose solidification, each PCR product was loaded into wells, and the reference well contained 10 Kb DNA ladder to determine the size of the PCR products. The gel was run in TBE buffer for 40 min at 180 volts and 210 mA and then visualized with the UV imager. 

### 4.2. Microliter Plate Assay

The attachment phase of biofilm formation was employed, using a modified microliter plate assay [24,47,48]. Briefly, each *P. aeruginosa* isolate was grown in tryptic soy broth at 37 °C overnight to allow biofilm formation. The bacterial culture was centrifuged at 4500 rpm for 15 min and washed twice with physiological buffer solution (PBS), and the pellet was suspended in Luria–Bertani broth. The optical density (OD_570_) of the bacterial suspension was adjusted to 0.1–0.2. In a 96-microtiter plate, a 180 μL LB broth was dispensed in each well, and 150 μL standardized bacterial culture (approximately 1.5 × 10^8^ CFU/mL) was added and mixed. The microplate was sealed and incubated at 37 °C for 24 h. The microplate was washed gently three times with sterile distilled water then dried. A quantitative evaluation of the biofilm formation was conducted by staining with crystal violet. To each well, 200 μL of 0.2% crystal violet stain was added, and the microliter plate was incubated at room temperature for 15 min. The plate was washed to remove the excess stain, and 100 μL of 95% ethanol was added and thoroughly mixed before the absorbance was read at 570 nm using a 96 well-spectrophotometer at 520 nm (Shimadzu-1700, Kyoto, Japan). The experiment was performed in triplicate, and the data are presented as average values. The values of the negative control (ODc) and the isolate (ODi) were determined according to the formula below [25]:

ODc = average ODc + (3 x standard deviation (SD) of ODc)

ODi = average of ODi − Odc

Based on the above calculations, the strength for biofilm formation was classified into four categories [25]:
Strongly adherent 4ODc < ODModerately adherent 2ODc < OD ≤ 4ODcWeakly adherent ODc < OD ≤ 2 ODcNon-adherent ODi ≤ ODc

### 4.3. Statistical Analysis

All bioassays were performed in triplicate, and data are presented as mean values with or without standard deviation (SD). The difference between the readings of the control the and sample was analyzed using Student’s t-test, and it was considered significant when *p* < 0.05.

## 5. Conclusions

This study highlights the importance of the molecular detection of *P. aeruginosa* in clinical settings. It strongly recommends using a molecular-based assay targeting preserved regions of *P. aeruginosa* genes to allow effective treatment, especially in older and immunocompromised patients. However, if conventional methods remain the only option for detection in healthcare practices, it is recommended to evaluate multiple signature virulence factors, including pigment production and/or biofilm formation, to avoid misidentification with other Gram-negative bacteria.

## Figures and Tables

**Table 1:** The concentration of pyocyanin produced by nine isolates validated as *P. aeruginosa*.

Isolate Producing Pyocyanin	Source of Isolate	Absorption at 520 nm	Conc. Pyocyanin (μg/mL)
1	Sputum	2.135	36.448
2	Ear	0.802	13.692
3	Wound	0.200	3.414
4	Wound	0.010	0.171
5	Urine	0.013	0.222
6	Urine	0.017	0.290
7	Skin	0.120	2.048
8	Skin	0.100	1.707
9	Skin	0.094	1.605

## Data Availability

Data are contained within the article.
